# Underwater endoscopic retrograde direct cholangioscopy facilitating biliary cannulation of intradiverticular duodenal papilla

**DOI:** 10.1055/a-2718-4893

**Published:** 2025-11-06

**Authors:** Shan-Shan Hu, Xiao-Gang Liu, Wei-Hui Liu

**Affiliations:** 1 Department of Gastroenterology and Hepatology, Sichuan Provincial Peopleʼs Hospital, School of Medicine, University of Electronic Science and Technology of China, Chengdu, China

Cannulation of intradiverticular papillae presents a common challenge in endoscopic retrograde cholangiopancreatography (ERCP), as the papilla is often situated within a diverticulum. Its concealed position, narrow orifice, and susceptibility to traction deformation or being obscured by overlying food debris all contribute to difficult exposure and relatively low cannulation success rates 1 . At the same time, the duodenoscope encounters difficulty in achieving smooth access to the diverticulum, compromising clear visualization of and proper alignment with the papillary orifice. Moreover, forcible insertion into the diverticulum would unavoidably elevate procedural risks. Recently, our team has successfully achieved cannulation of intradiverticular papillae by employing the innovative endoscopic retrograde direct cholangioscopy (ERDC) 2 3 technique under water. The key technological innovation lies in the conical transparent cap mounted on the distal end of a child scope, which serves as a direct-view endoscope. This configuration permits thorough visualization of the papillary orifice within the diverticulum and facilitates precise alignment through manipulation of four control knobs for successful cannulation.

A 65-year-old man underwent ERCP for choledocholithiasis. During endoscopic examination, the duodenal papilla was found to be situated on the lateral wall of a diverticulum, with its orifice oriented towards the diverticular lumen ( Fig. 1 ). The ERDC technique was selected for biliary cannulation. Initially, water was instilled into the diverticulum to reduce interference from intestinal fluid, food residue, and mucosal folds obscuring the papilla, thereby maintaining stable visualization ( Fig. 2 ). Subsequently, a transparent cap was affixed to the distal end of the cholangioscope to help it visualize and stabilize the papilla within the diverticulum ( Fig. 3 ). Under the direct vision of the cholangioscope, the biliary orifice was promptly located, enabling successful biliary cannulation and complete clearance of the choledocholithiasis ( Fig. 4 ; Video 1 ).

**Fig. 1:**
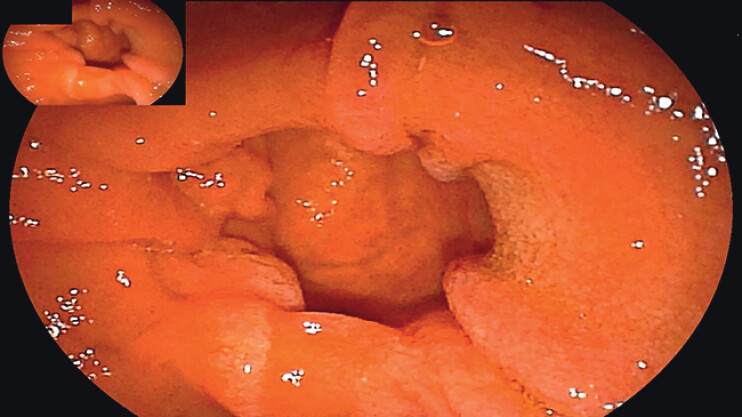
Intradiverticular papilla in a 65-year-old man with choledocholithiasis.

**Fig. 2:**
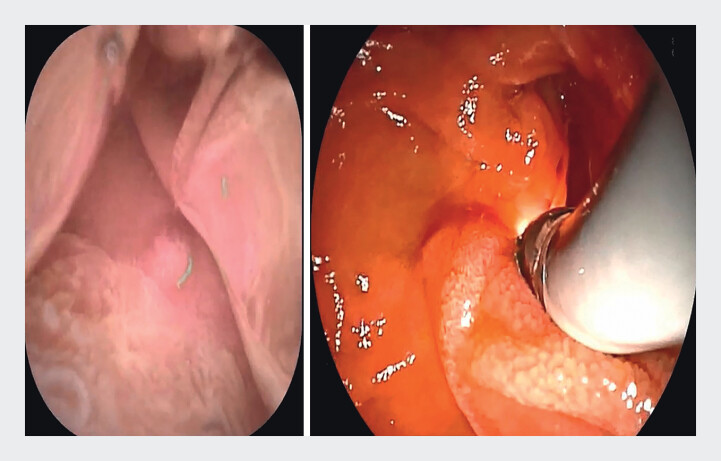
Endoscopic retrograde direct cholangioscopy (ERDC) carried out under water.

**Fig. 3:**
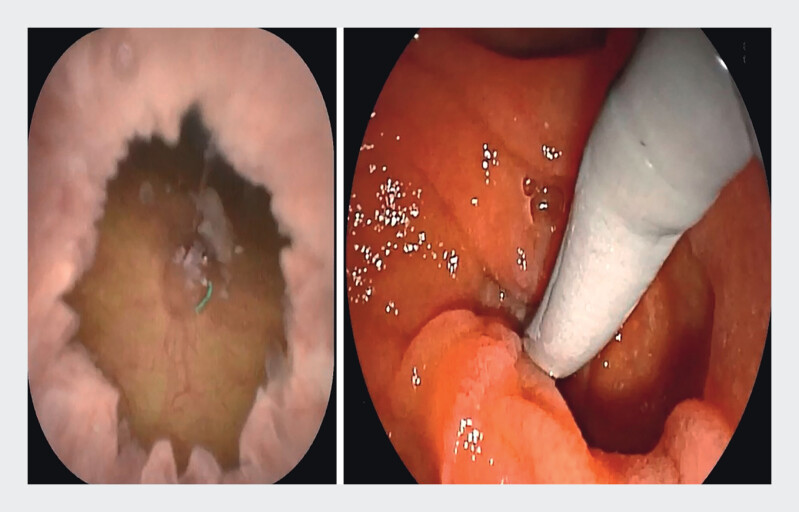
Bile duct cannulation under ERDC vision.

**Fig. 4:**
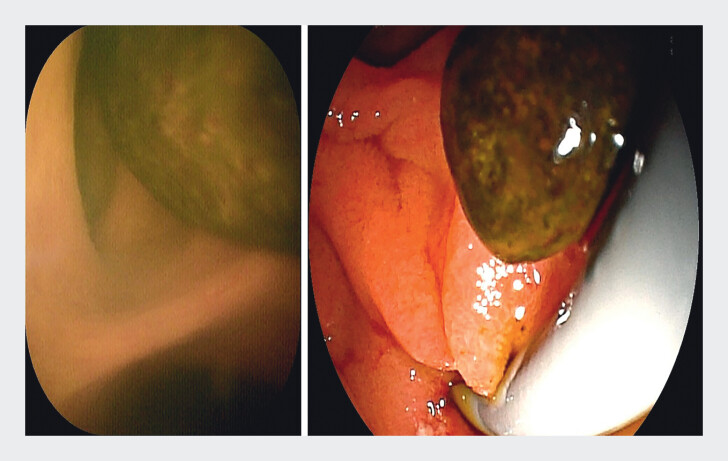
Lithotomy under ERDC vision.

The novel technique of endoscopic retrograde direct cholangioscopy provides a safe and effective approach for cannulation in patients with diverticular papillae.Video 1

Given the confined space of the diverticular lumen and the delicate mucosal tissue, conventional ERCP instrumentation carries considerable risks when performing blind manipulation. By comparison, the child scope fitted with a conical cap is ideally suited to such anatomical configurations, offering unobstructed direct visualization that ensures both procedural efficacy and operational safety during cannulation.

Endoscopy_UCTN_Code_TTT_1AR_2AH

## Notes

Endoscopy E-Videos https://eref.thieme.de/e-videos
*E-Videos* is an open access online section of the journal *Endoscopy* , reporting on interesting cases and new techniques in gastroenterological endoscopy. All papers include a high-quality video and are published with a Creative Commons CC-BY license. Endoscopy E-Videos qualify for HINARI discounts and waivers and eligibility is automatically checked during the submission process. We grant 100% waivers to articles whose corresponding authors are based in Group A countries and 50% waivers to those who are based in Group B countries as classified by Research4Life (see: https://www.research4life.org/access/eligibility/ ). This section has its own submission website at https://mc.manuscriptcentral.com/e-videos .

## References

[1] Facciorusso A Ramai D Gkolfakis P Comparative efficacy of different methods for difficult biliary cannulation in ERCP: systematic review and network meta-analysis Gastrointest Endosc 2022 95 60 7.1E13 10.1016/j.gie.2021.09.010 34543649

[2] Liu WH Huang XY Hu X Initial experience of visualized biliary cannulation during ERCP Endoscopy 2023 55 1037 1042 10.1055/a-2113-8952 37339664

[3] Liu WH Huang XY Zhang RY From darkness to brightness: the cholangioscopy-guided selective biliary cannulation with the help of transparent cap during ERCP Endoscopy 2023 55 E320 E321 10.1055/a-1981-2503 36513111 PMC9833945

